# Ergosterol-depleted clinical isolates of *Nakaseomyces glabratus* can develop multi-drug resistance without severe fitness defects or attenuated virulence in an invertebrate infection model

**DOI:** 10.1128/mbio.02731-25

**Published:** 2026-05-20

**Authors:** Alexander M. Aldejohann, Nadja Thielemann, Aina Martinez Zurita, Christoph Müller, Tom Gräfenhan, Richard Kriz, Heimo Lagler, Isabell S. Behr, Nathalie Reus, Annika Schöninger, Grit Walther, Lena-Marie Mazan, Hannah Wilhelm, Birgit Willinger, Christina A. Cuomo, Oliver Kurzai, Ronny Martin

**Affiliations:** 1 University of Würzburg, Institute for Hygiene and Microbiology, Würzburg, Germany; 2 Infection Control and Antimicrobial Stewardship Unit, University Hospital Würzburg 676759, Würzburg, Germany; 3 Infectious Disease and Microbiome Program, Broad Institute of MIT and Harvard, Cambridge, Massachusetts, USA; 4 Department of Pharmacy-Center for Drug Research, Ludwig Maximilians University Munich, Munich, Germany; 5 Core Unit Systems Medicine, University of Würzburg, Würzburg, Germany; 6 Division of Infectious Diseases and Tropical Medicine, Department of Medicine I, Medical University of Vienna, Vienna, Austria; 7 Section Biomedical Science, Health Sciences, University of Applied Science Campus Wien, Vienna, Austria; 8 National Reference Center for Invasive Fungal Infections, Leibniz Institute for Natural Product Research and Infection Biology-Hans Knoell Institute, Jena, Germany; 9 Division of Clinical Microbiology, Department of Laboratory Medicine, Medical University of Vienna, Vienna, Austria; 10 Department of Molecular Microbiology and Immunology, Brown University, Providence, Rhode Island, USA; 11 Research Group Fungal Septomics, Leibniz Institute for Natural Product Research and Infection Biology-Hans Knoell Institute, Jena, Germany; The University of Texas Health Science Center at Houston, Houston, Texas, USA

**Keywords:** *Nakaseomyces glabratus*, amphotericin B, ergosterol depletion, multidrug resistance

## Abstract

*Nakaseomyces glabratus* (formerly *Candida glabrata*) is a leading cause of invasive candidiasis and rapidly develops antifungal drug resistance during treatment. An increasing number of clinical isolates show reduced susceptibility to echinocandins and azoles, leaving amphotericin B (AMB) as a last therapeutic option. Resistance of *N. glabratus* to this drug is rare, and its underlying mechanisms are still not fully understood. Here, we describe two independent multidrug-resistant bloodstream isolates displaying resistance to AMB and anidulafungin (ANF), as well as a reduced susceptibility to azoles. We performed whole-genome sequencing and sterol profiling on nine clinical *N. glabratus* isolates, which were resistant to ANF and displayed resistance or low susceptibility to fluconazole (FLU) and AMB. We identified loss-of-function mutations in the genes *ERG3* and *ERG4*, which could be linked to ergosterol depletion and AMB resistance. The transcriptional response of the reference strain CBS138 and an AMB^R^ + ANF^R^ isolate was analyzed by RNA-seq, revealing that ergosterol depletion also contributed to upregulation of *ERG* and ABC transporter genes, which might explain the low FLU susceptibility. Surprisingly, the AMB^R^ isolates displayed severe fitness defects, and one of them was fully virulent in a *Galleria mellonella* infection model. Our results indicate that ergosterol depletion in *N. glabratus* leads to AMB resistance without affecting fitness or virulence.

**IMPORTANCE:**

The major human fungal pathogen *Nakaseomyces glabratus* is well known for its fast development of antifungal drug resistance, especially against commonly used azoles. However, it can also acquire resistance to echinocandins, leading to multidrug resistance (MDR) and leaving amphotericin B (AMB) as the last therapeutic option. AMB resistance is rare, mainly caused by ergosterol depletion, and is normally associated with severe fitness costs for the pathogen. However, we found *N. glabratus* bloodstream isolates with stable AMB resistance without apparent fitness and virulence defects. The underlying ergosterol depletion contributed to low azole susceptibility and was associated with anidulafungin resistance. These findings demonstrate how fast MDR can evolve in *N. glabratus* and underline the need for close resistance monitoring.

## INTRODUCTION

Invasive candidiasis is a life-threatening fungal infection caused by yeasts, including *Candida albicans* and *Nakaseomyces glabratus*, and comprises bloodstream infections, but also dissemination to organs like the liver and kidney, accounting for approximately 1.5 million cases per year with attributable mortality of up to 60% (1–3). While *C. albicans* is the most virulent and best studied pathogen and still accounts for approximately 50% of systemic *Candida* infections, non-*albicans Candida* species are of increasing importance as they often develop antifungal drug resistance or even multidrug-resistant (MDR) (1, 4). The recommended first-line treatment for systemic *Candida* infections is echinocandins (5, 6). They bind the catalytic subunit of the β-(1,3)-D-glucan synthetase and inhibit the β-(1,3)-D-glucan biosynthesis, leading to disruption of cell wall integrity and osmotic imbalance (7). Echinocandin resistance is mainly caused by point mutations in the hot spot regions of the *FKS* genes, which encode the enzyme’s catalytic subunit (8). Echinocandin resistance in *N. glabratus* is rare, but has increased in recent years (9). Due to the intrinsically low susceptibility of *N. glabratus* to azoles and a high proportion of azole-resistant isolates, acquisition of echinocandin resistance often results in MDR, leaving liposomal amphotericin B (AMB) as an indispensable option, despite severe side effects for the patients, such as high nephrotoxicity or serum electrolyte changes (1, 5, 6). AMB binding to ergosterol leads to either the formation of small ion channels or ergosterol extraction from the cell membrane (10–12). AMB resistance is still very rare among *Candida* species. Ergosterol depletion appears to be the major resistance mechanism but is often associated with high fitness costs (7, 13, 14).

Here, we describe AMB resistance in two independent clinical *N. glabratus* isolates and show that combined mutations in *ERG3* and *ERG4* are responsible for the resistance but do not result in fitness defects. One of the isolates also displayed decreased susceptibility to anidulafungin (ANF) and azoles. The latter was Pdr1-dependent and likely triggered by ergosterol depletion. Thus, ergosterol depletion associated with AMB resistance can directly result in MDR phenotypes in *N. glabratus*.

## RESULTS

### *ERG3* mutations are enriched among MDR isolates

**Fig 1:**
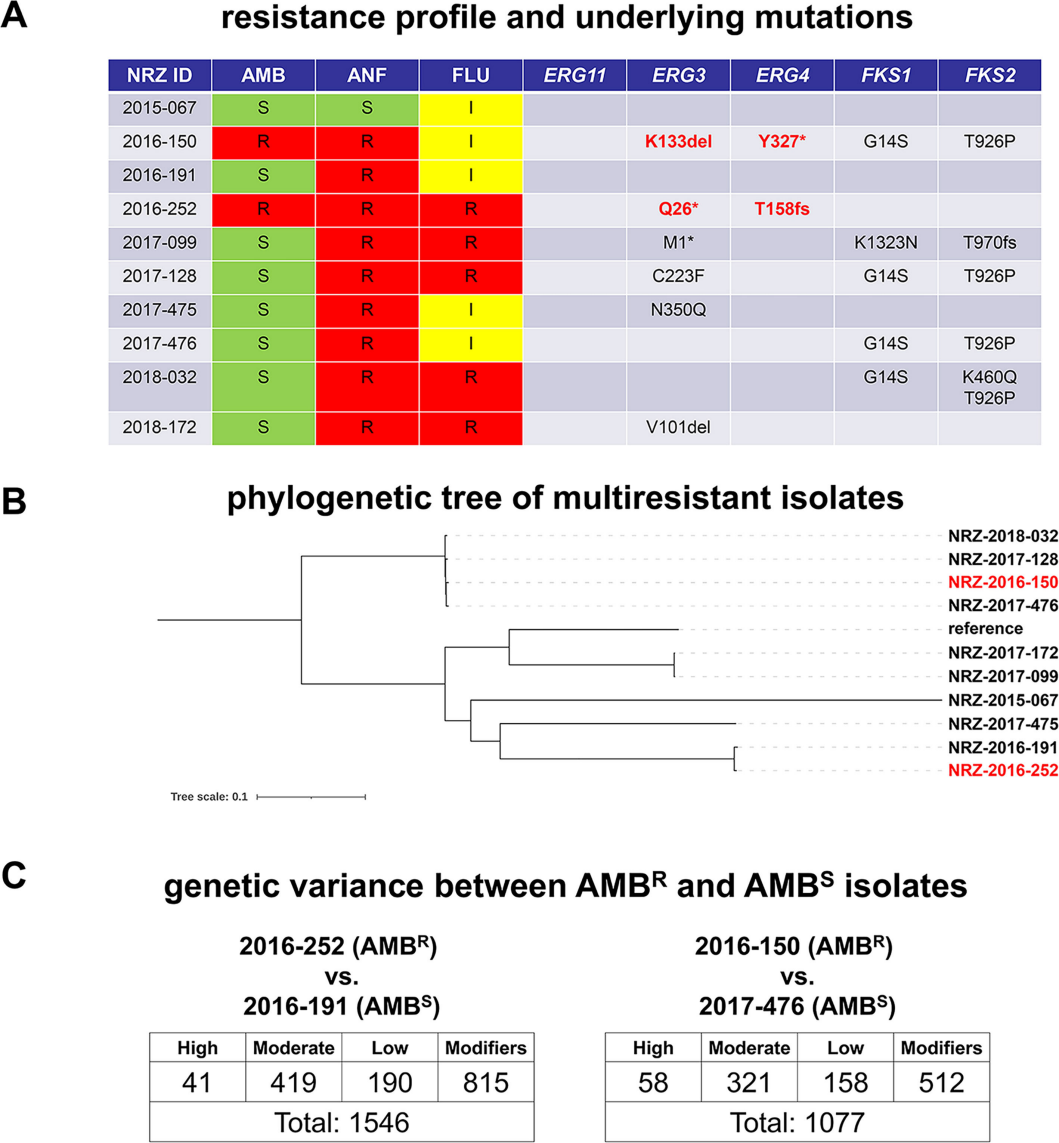
Resistance profile and genetic characteristics of MDR *N. glabratus* isolates. (**A**) Summary of antifungal susceptibility testing (AFST) and underlying mutations in possible resistance genes. AFST was performed with broth microdilution and based on EUCAST clinical breakpoints, the isolates were determined as susceptible (S), increased dose dependent (I), or resistant (R). Shown mutations in the indicated genes were identified by WGS. (**B**) WGS-based phylogenetic tree of the examined *N. glabratus isolates*. The AMB^R^ isolates are marked in red. (**C**) Genetic diversity of AMB^R^ isolates NRZ-2016-252 and NRZ-2016-150 and their closest relatives NRZ-2016-191 and NRZ-2017-476.

We have previously analyzed the emergence of echinocandin-resistant clinical isolates of *N. glabratus* in Germany (9). Based on this work, we further examined strains, which displayed (i) resistance to the echinocandin ANF without harboring *FKS* hot spot mutations and (ii) additional resistance to either FLU or AMB (=MDR isolates, Fig. 1A). Whole-genome sequencing (WGS) was performed for all six identified MDR isolates and three isolates with isolated ANF resistance (ANF^R^) and one control isolate (AMB^S^, ANF^S^, and FLU^I^). The MDR strains formed no clear cluster but showed enrichment of *ERG3* mutations (Fig. 1A and B). The two AMB^R^ strains were genetically unrelated. Their respective closest relatives were AMB^S^ and displayed a high degree of genetic differences compared to the AMB^R^ strains (Fig. 1B and C). We identified putative loss-of-function mutations in the *ERG3* genes of the strains NRZ-2017-099 (M1*), NRZ-2016-252 (Q26*), and NRZ-2016-150 (K133^del^) (Fig. 1A). The latter two strains also displayed putative loss-of-function mutations in the *ERG4* genes: T158^fs^ and Y327* (Fig. 1A).

We identified several *FKS* mutations in our strains. Interestingly, the G14S mutation in *FKS1* and the T926P mutation in *FKS2* were simultaneously found in four strains (Fig. 1A). However, only the isolate NRZ-2017-099 harbored two mutations, which might explain ANF resistance: K1323N in *FKS1* and T970fs in *FKS2* (Fig. 1A). Despite displaying ANF resistance, no *FKS* mutations were identified in NRZ-2016-252, NRZ-2016-191, and NRZ-2017-475 (Fig. 1A).

### Cell wall composition of MDR isolates

**Fig 2:**
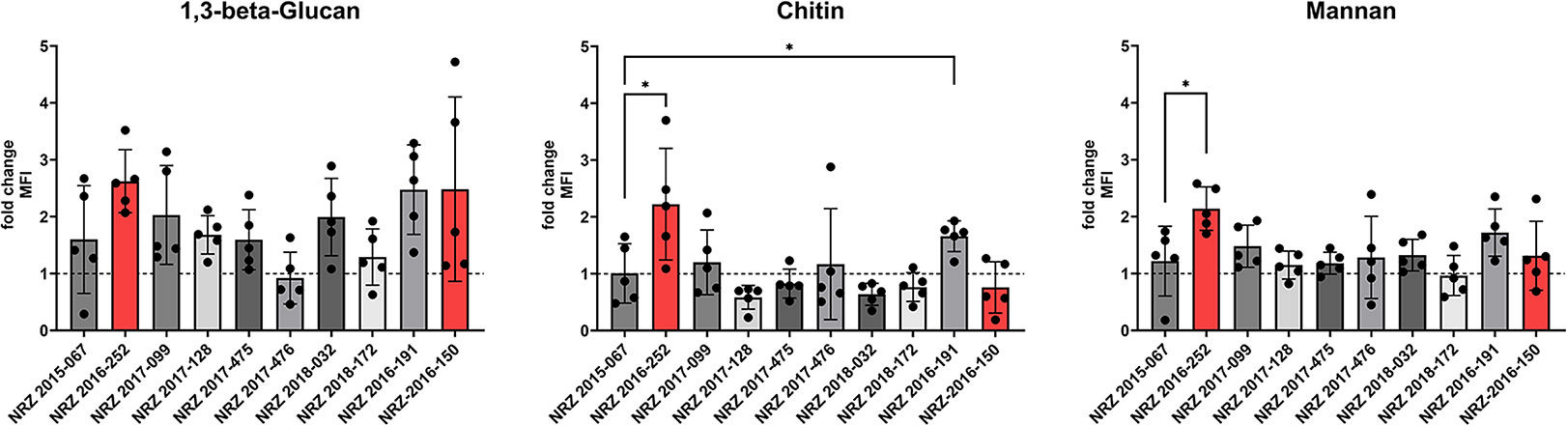
Cell wall composition of MDR *N. glabratus* isolates. The indicated *N. glabratus* strains were grown in YPD at 37°C, harvested, and then stained for chitin, β-1,3-D-glucan, and mannan. The amounts of the three cell wall components from five independent biological experiments were measured by flow cytometry. The values were normalized against the values of the control strain CBS138. The AMB^R^ isolates are marked with red columns. Asterisks indicate significant differences in the cell wall components between the indicated strains and the susceptible isolate NRZ-2015-067 (*P* ≤ 0.05, two-tailed, unpaired Student’s *t* test).

Therefore, we used flow cytometry to measure the amounts of chitin, glucan, and mannan within the isolates, as an altered composition is sometimes associated with echinocandin resistance. After normalization against the reference strain CBS138, we compared all ANF^R^ isolates with the ANF^S^ isolate NRZ-2015-067. While no significant differences were detectable for β-(1,3)-D-glucan, we observed that the AMB^R^ + ANF^R^ isolate NRZ-2016-252 had significantly more chitin and mannan in its cell wall than the ANF^S^ isolate NRZ-2015-067 (Fig. 2). The chitin content in NRZ-2016-191 was also higher than in the control strain (Fig. 2). Interestingly, NRZ-2016-150, the other AMB^R^ + ANF^R^ isolate, displayed no significant differences to the susceptible strain NRZ-2015-067 (Fig. 2).

### Ergosterol depletion correlates with resistance to AMB

**Fig 3:**
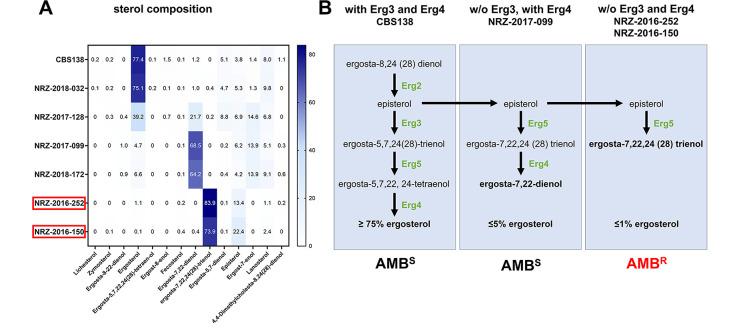
Sterol composition analysis of CBS138 and the clinical isolates. (**A**) Sterol composition of the indicated strains was analyzed by gas chromatography-mass spectrometry (GC-MS). Shown are the percentages of the single sterols in the indicated strains. Red rectangles mark the AMB^R^ isolates. (**B**) Illustration of the effects of the absence of either Erg3 and/or Erg4 in AMB^S^ and AMB^R^ strains. Shown are branches of the ergosterol biosynthesis pathway in the absence of Erg3 and/or Erg4, leading to the production of non-physiological precursors.

We additionally examined the sterol composition of the strains that exhibited resistance to AMB or azoles. With a percentage of 77%, ergosterol was the main sterol in the reference strain CBS138 (Fig. 3A; Table S1). The *ERG3* mutations M1*, V101del, and C223F in the isolates NRZ-2017-099, NRZ-2018-172, and NRZ-2017-128 were associated with decreased ergosterol levels and the accumulation of ergosta-7,22-dien-3β-ol and ergost-7-en-3β-ol, indicating that the function of the sterol C5-desaturase Erg3 is disturbed or lost (Fig. 3A). In all three strains, the *ERG3* mutations correlated with an additional FLU resistance. The AMB^R^ + ANF^R^ isolates NRZ-2016-252 and NRZ-2016-150 had extremely low ergosterol concentrations (0.1%–1%) while ergosta-7,22,24(28)-trien-3β-ol increased up to 85% (Fig. 2; Table S1). This is only possible if neither Erg3 nor Erg4 work properly, fitting to the identified mutations in both strains (Fig. 1A and 3B). Strains with low ergosterol concentration of 4%–6% are still susceptible to AMB, indicating that complete depletion is required for AMB resistance (Fig. 3B).

### Absence of functional *ERG3* and *ERG4* induces resistance to AMB and ANF

**Fig 4:**
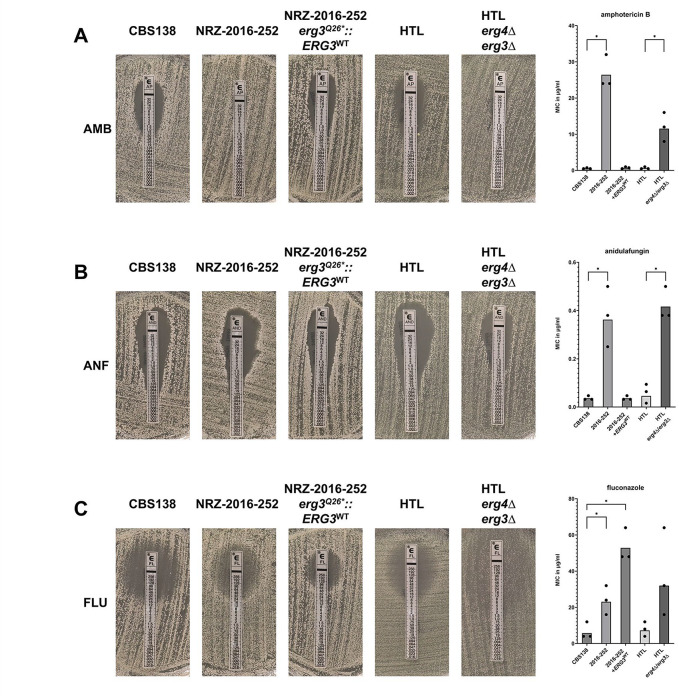
Absence of *ERG3* and *ERG4* is required for resistance to AMB and ANF. (**A–C**) Integration of a wild-type *ERG3* allele into the AMB^R^ isolate NRZ-2016-252 increased susceptibility to AMB and ANF but not to FLU. Deletion of *ERG4* and *ERG3* in the susceptible *N. glabratus* HTL background strain results in resistance to AMB and ANF only. The indicated strains were plated onto RPMI1640 medium, and E-test stripes for AMB (**A**), ANF (**B**), and FLU (**C**) were applied. The plates were then grown for 48 h at 37°C before pictures were taken. The shown pictures are representative of three independent biological experiments. (**A–C**) The geometric means of MICs to the respective antifungal drug are shown on the right side. Asterisks indicate significant differences between the indicated strains (∗*P* ≤ 0.05, two-tailed, unpaired Student’s *t* test).

We hypothesized that the simultaneous loss of function in Erg3 and Erg4 enzymes causes AMB resistance and also leads to resistance against ANF and low susceptibility to FLU. To confirm this, we replaced the *ERG3*^Q26*^ allele in NRZ-2016-252 with the wild-type allele of CBS138. The resulting mutant became susceptible to AMB and ANF (Fig. 4A and B). Surprisingly, the strain was FLU^R^, maybe caused by the still present *ERG4*^T158fs^ mutation (Fig. 4C). Additionally, we deleted *ERG3* and *ERG4* in the background strain HTL. The resulting double mutant displayed significant AMB and ANF resistance to the background strain, similar to NRZ-2016-252 (Fig. 4A and B). The susceptibility to FLU also decreased. However, while we observed a significantly lower susceptibility of NRZ-2016-252 to FLU compared to CBS138, this was not the case between HTL and *erg4*∆/*erg3*∆ (Fig. 4C).

### Pdr1 links ergosterol depletion and low susceptibility to FLU

**Fig 5:**
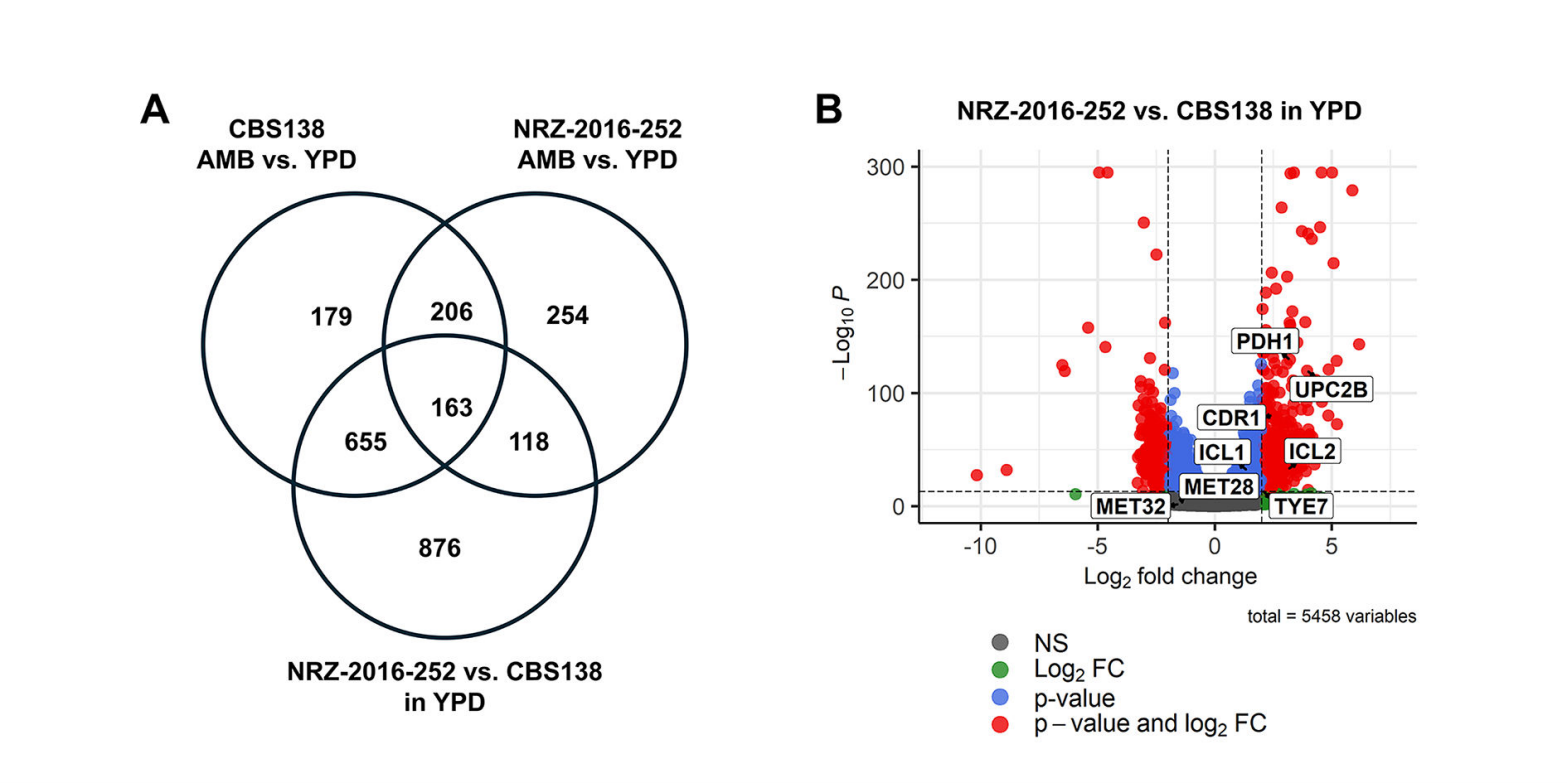
Transcriptional response of AMB^S^ and AMB^R^ strains to AMB. The AMB^S^ strain CBS138 and the AMB^R^ strain NRZ-2016-252 were grown for 1 h in YPD with 1 µg/mL AMB (AMB) or without 1 µg/mL AMB (YPD) prior to RNA isolation. For each condition, three independent biological experiments were performed. (**A**) Comparison of upregulated genes in CBS138 and NRZ-2016-252 in AMB and of genes upregulated in NRZ-2016-252 compared to CBS138 in YPD. Example genes upregulated in NRZ-2016-252 compared to CBS138 in the presence or absence of AMB are shown in the box. (**B**) Volcano plot of differentially expressed genes in NRZ-2016-252 compared to CBS138 after 1-h growth in YPD at 37°C.

To better understand the biology of AMB^R^ strains, we examined the transcriptomes of the AMB^S^ strain CBS138 and the AMB^R^ isolate NRZ-2016-252 after 1-h incubation in YPD with or without 1 µg/mL AMB at 37°C. In total, 1,203 genes were differentially expressed in CBS138 in response to AMB (Fig. 5A; Table S2). Interestingly, 68% of them were differentially expressed in the AMB^R^ strain NRZ-2016-252 in the absence of AMB, indicating that this strain is already well adapted to AMB (Fig. 5A; Table S2). Among the upregulated genes in the AMB^R^ strain were stress-related genes (*UPC2B, TYE7*, *ICL1*, *ICL2*, *HSP12,* and *RAD27*), efflux pump genes (*CDR1*, *PDH1*, *FLR1,* and *FLR2*), and ergosterol biosynthesis genes (Fig. 5B; Table S2). The latter two groups are known targets of the transcription factor Pdr1 (15, 16). Especially the upregulation of the efflux pump genes might explain the low FLU susceptibility of NRZ-2016-252. Deletion of the *PDR1* gene caused a dramatic decline of *CDR1, FLR1,* and *PDH1* transcription in NRZ-2016-252 (Fig. S1A). Compared to NRZ-2016-252, the *pdr1*∆ derivative was extremely susceptible to FLU, indicating that upregulation of *CDR1* and *PDH1* in NRZ-2016-252 was required for the low FLU susceptibility of NRZ-2016-252 (Fig. S1B). NRZ-2016-252 *pdr1*∆ remained AMB and ANF resistant, illustrating that Pdr1 was not required for resistance to these drugs (Fig. S1B).

### AMB^R^ isolates display no severe fitness defects or attenuated virulence

**Fig 6:**
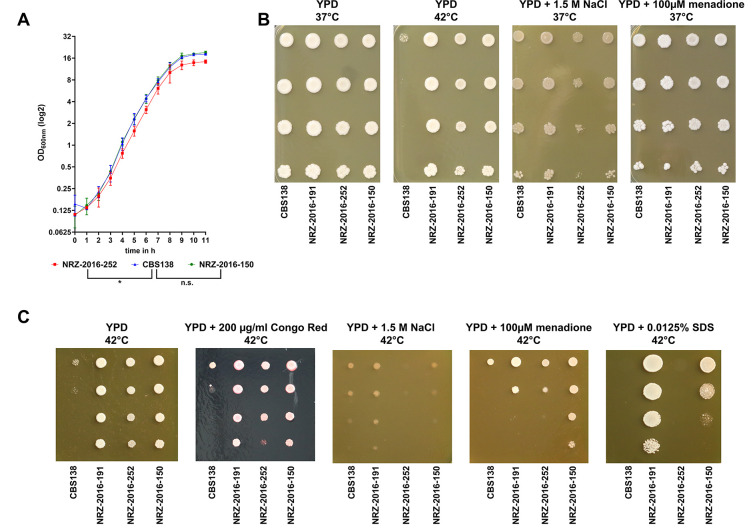
AMB^R^ isolates show no fitness defects under single but under combinatory stress conditions. (**A**) Growth curve of CBS138, NRZ-2016-252, and NRZ-2016-150 grown in YPD medium at 37°C for 11 h. The optical density of the three strains was measured by photometry at 600 nm. Shown are the average values and standard deviations from three independent biological experiments. Asterisk indicates significant differences in growth (Friedman testing, followed by Dunn’s multiple comparison test, *P* ≤ 0.05). (**B**) The indicated *N. glabratus* strains were grown on YPD with or without 1.5 M sodium chloride or 100 µM menadione to induce osmotic and oxidative stress. From top to bottom, 5 µL of 1 × 10^8^, 1 × 10^6^, 1 × 10^4^, and 1 × 10^2^ cells/mL were spotted onto the plates. They were incubated at 37°C and 42°C for 3 days prior to photography. (**C**) The same *N. glabratus* strains were grown on YPD at 42°C with or without 200 µg/mL Congo Red to initiate cell wall stress, 1.5 M NaCl for osmotic stress, 100 µM menadione for oxidative stress, and 0.0125% SDS for cell membrane stress. Cells were spotted onto the plates as described in panel **B**. The plates were grown for 3 days prior to photography. Plates shown in panels **B** and **C** are representative of three independent biological experiments.

As AMB resistance is often linked to profound fitness defects, we examined the growth dynamics of the AMB^R^ isolates under different conditions. We first compared their growth dynamics in YPD medium at 37°C to the reference strain CBS138. Growth of NRZ-2016-252 was slightly but significantly reduced, while there were no differences between NRZ-2016-150 and CBS138 (Fig. 6A). Despite these differences, the doubling times of all three strains were similar to each other (CBS138: 57.8 ± 4.5 min; NRZ-2016-150: 60.4 ± 2.3 min; NRZ-2016-252: 59.8 ± 2.2 min). Spotting assays on solid YPD, minimal medium, and RPMI1640 did not display visible growth differences between the two AMB^R^ strains and CBS138 (Fig. S2A). We then tested the growth of these strains, the AMB^S^ isolate NRZ-2016-191 and the reference strain CBS138, under several stress conditions. Despite the ergosterol depletion, both AMB^R^ isolates showed good stress resilience at 37°C and robust growth at 42°C (Fig. 6B; Fig. S2). Only NRZ-2016-252 displayed a growth delay under osmotic stress conditions (1.5 M NaCl) and was also more susceptible to combined stressors such as 42°C and 1.5 M NaCl or 0.0125% SDS (Fig. 6B and C). However, these growth inhibitions were less pronounced in the other AMB^R^ isolate, NRZ-2016-150.

**Fig 7:**
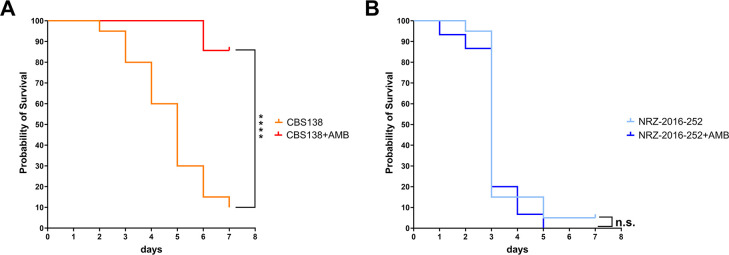
*N. glabratus* NRZ-2016-252 shows resistance against AMB under *in vivo* conditions. *Galleria mellonella* larvae were either infected with 2 × 10^7^ CBS138 cells (**A**) or NRZ-2016-252 cells (**B**). If required, infected larvae were treated with 5 mg AMB per kg body weight. 20 larvae, pooled from two independent biological experiments, were used for each condition and incubated for up to 7 days at 37°C. The evaluation of survival of the larvae, indicated by melanization and mobility, was first monitored 12 h post-infection and then periodically every 24 h. Survival rates were visualized by Kaplan-Meier plots. Asterisks indicate significant differences in survival rates (Mantel-Cox test, *P* ≤ 0.0001).

Finally, we analyzed the virulence of CBS138 and the AMB^R^ strain NRZ-2016-252 in an invertebrate infection model with *Galleria mellonella* larvae under AMB treatment. Without treatment, CBS138 killed the infected larvae within 7 days (Fig. 7A). However, after treatment with AMB, 86% of the larvae (12/14) survived the infection with CBS138 after 7 days (Fig. 7A). Larvae infected with the AMB^R^ isolate NRZ-2016-252 died within the first 4 days, independent from the addition of AMB, validating the observed *in vitro* resistance to the drug under *in vivo* conditions (Fig. 6B). In our settings, NRZ-2016-252 was as virulent as CBS138, illustrating that ergosterol depletion did not negatively affect the virulence of this strain in an invertebrate infection model.

## DISCUSSION

Our study showed that loss-of-function mutations in the *ERG3* and *ERG4* genes led to ergosterol depletion and consequently AMB resistance, similar to previous findings for *Clavispora lusitaniae* and *Candida auris* (syn. *Candidozyma auris*) (17, 18). Absence of functional Erg3 and Erg4 resulted in a massive shift from ergosterol to ergosta-7,22,24(28)-trien-3βol in the two AMB^R^ isolates, which was also reported for AMB^R^ *C. auris* strains (18). This understudied sterol has a similar binding affinity to liver X receptors (LXR) as ergosterol and is often enriched in ergosterol-lacking fungal strains (19–21). The primary effect of ergosterol depletion is AMB resistance, as the drug can no longer bind to its target ergosterol. Additionally, it induced a transcriptional adaptation against AMB even in its absence, including the upregulation of Pdr1-controlled genes *CDR1* and *PDH1*. We presume that the increased expression of these ABC transporter genes leads to increased efflux pump activity and therefore to reduced susceptibility to FLU, similar to previous observations (15, 16). Additionally, the transcriptome data of AMB^R^ strains might be used in the future to identify candidate genes for a resistance mechanism against AMB other than ergosterol depletion. The observed association between AMB^R^ and ANF^R^ without underlying *FKS* hot spot mutations could not be fully explained. Changes in the cell wall and the cell membrane caused by the absence of ergosterol may reduce the accessibility of the β-1,3-D glucan synthase for anidulafungin. Similar to previous work (22), our strains were susceptible to micafungin, either caused by a higher affinity or less interaction with the altered cell membrane.

Strikingly, AMB^R^ isolate NRZ-2016-252 was fully virulent in a *Galleria mellonella* infection model. Its overall *in vitro* and *in vivo* fitness contradicts the hypothesis that acquisition of AMB resistance is associated with high fitness costs (14, 18). Especially, the bloodstream was previously discussed as an environment too hostile for the survival of AMB-resistant strains (14); however, our two AMB^R^ isolates were obtained from blood cultures. We presume that the acquisition of AMB resistance alone made the strains not more susceptible to the harsh conditions within the bloodstream. This is in accordance with the findings from the stress response assays. The AMB^R^ isolates showed surprisingly high resilience against environmental stressors. The transcriptome of NRZ-2016-252 also showed that this AMB^R^ isolate is well adapted to environmental stress. Only the combination of 42°C with osmotic or oxidative stress led to growth defects, especially in NRZ-2016-252, while NRZ-2016-150 was less affected. Therefore, we conclude that even isolates with a full depletion of ergosterol can display normal fitness and virulence. However, the underlying mechanisms remain unclear and should be studied in the future. One possibility could be suppressor mutations that might have bypassed some defects caused by ergosterol depletion, which is supported by the fact that the *erg4*∆/ *erg3*∆ deletion mutant in the HTL background displayed severe growth defects already under normal growth conditions.

We described *N. glabratus* bloodstream isolates with stable AMB resistance without apparent fitness and virulence defects. In combination with the intrinsically low susceptibility to azoles and the emerging echinocandin resistance, these findings underline the threat of an increasing MDR and extensively drug resistance (XDR) in this major human fungal pathogen. Close resistance monitoring is therefore urgently needed.

## MATERIALS AND METHODS

### Strains and media

All strains used in this study are listed in Table S3. They were routinely grown in YPD medium (20 g/L glucose, 10 g/L yeast extract, and 20 g/L peptone) at 37°C unless otherwise indicated.

### Antifungal drug susceptibility testing

AFST was either performed with EUCAST-based broth microdilution (23) or with Etests (Biomérieux) according to the manufacturer’s instructions.

### DNA isolation and WGS

The ZR Fungal/Bacterial DNA MiniPrep kit (Zymo Research, Irvine, CA, USA) was used to extract fungal genomic DNA. Genomic libraries were constructed and barcoded using the NEBNext Ultra DNA Library Prep kit for Illumina (New England Biolabs, Ipswich, MA, USA) and then sequenced using the Illumina platform. Paired-end reads were aligned to the *N. glabratus* genome of reference strain CBS138 (GenBank accession GCA_000002545.2) using BWA mem v0.7.12. Variants were then identified using GATK v4.1.4.1 (24) using the haploid mode and GATK tools MarkIlluminaAdapters, MarkDuplicates, AddOrReplaceReadGroups, HaplotypeCaller for both SNPs and indels, CombineGVCFs, GenotypeGVCFs, GatherVCFs, SelectVariants, and Variant Filtration. Sites were filtered with Variant Filtration using “QD < 2.0 | FS > 60.0 | MQ < 40.0.” Genotypes were filtered if the minimum genotype quality <50, percent alternate allele <0.8, or depth <10 (25). The variant calling pipeline is detailed in the following github repository: https://github.com/broadinstitute/fungal-wdl/tree/master/gatk4. Genomic variants were annotated, and the functional effect was predicted using SnpEff v4.3T (26). A phylogenetic tree was generated from variant sites that had less than 10% of ambiguous samples using FastTree v2.1.8 (27). Counts of unique variants differing between related isolates were obtained using the bcftools view command to select for isolates of interest, followed by bcftools filter with filtering parameters “-i 'AC=1 & AN=2”.

### Cell wall composition analysis

Staining of the *N. glabratus* cell wall components was performed according to a previously published protocol (28). We have used an anti-β-1,3-glucan antibody (1 mg/mL, Biosupplies) for the primary staining of β-1,3-glucan, ConA647 (5 mg/mL, Sigma Aldrich) for the primary staining of mannan, and WGA-FITC (2 mg/mL, Sigma Aldrich) for the primary staining of chitin. Secondary staining was performed with a goat anti-mouse PE-Cy7 antibody (0.2 mg/mL, BioLegend). Flow cytometry was conducted with a CytoFlex (Beckman Coulter), and for each measurement, 10,000 events were counted. Data analysis was performed with FlowJo v10.10.0 software. Fluorescence intensities of the clinical isolates were normalized against the fluorescence intensity of the control strain CBS138. All ANF^R^ isolates were compared with the ANF^S^ isolate NRZ-2015-067. Data from five independent experiments were analyzed with a two-tailed, unpaired Student’s *t*-test, and *P* values ≤ 0.05 were regarded as statistically significant.

### Measurement of sterol components

The sterol composition of clinical *N. glabratus* isolates was determined by GC-MS as previously described (29).

### Plasmid construction

To construct a deletion cassette for *N. glabratus ERG4*, a 1,000 bp region upstream of the starting codon was amplified with primers 5′CgERG4-PstIoverlap and 5′CgERG4-ScLEU2p-OL (Table S4). A 1,000 bp region downstream of the *CgERG4* stop codon was amplified with primers 3′CgERG4-PstIoverlap and 3′CgERG4-ScLEU2t-OL (Table S4). In addition, the *Saccharomyces cerevisiae* gene *LEU2* was amplified from genomic DNA with primers ScLEU2p-CgERG4-OL and ScLEU2-CgERG4-OL (Table S4). The resulting three PCR products, which contained overlapping sequences to each other, were then ligated into a *Pst*I-restricted pBluescript (Agilent) by using the NEBuilder HiFi DNA assembly Cloning kit (New England Biolabs). The constructed plasmid was named pSK-CgERG4-ScLEU2. The plasmid pSK-CgERG3-ScHIS3 was constructed in a similar way, using the *ScHIS3* gene and a *Sac*II-restricted pSK Bluescript. The primers used for plasmid construction are listed in Table S4. For the construction of the *PDR1* deletion cassette, we have amplified the *NAT1* resistance gene from the plasmid pSK-SCH9-NAT1 (30) and the 1,000 bp homology regions for integration into the *CgPDR1* locus from genomic CBS138 DNA. Primers used for these amplification steps are listed in Table S4. The resulting PCR products were cloned into *Sac*II-restricted pBluescript, leading to plasmid pSK-CgPDR1-NAT1. In a similar way, *ERG3* from CBS138, including promoter and terminator regions, was amplified from genomic CBS138 DNA and cloned into *Sac*II-restricted pBluescript, leading to plasmid pSK-CgERG3^WT^-NAT1. The used primers are shown in Table S4. All plasmids are listed in Table S5.

### Strain construction

The deletion cassettes CgPDR1-NAT1, CgERG4-ScLEU2, and CgERG3-ScHIS3 were amplified from the plasmids by using the primers pSK forward-2 and pSK reverse (Table S4). For the transformation of the deletion cassettes into *N. glabratus,* we have used the lithium acetate protocol as previously described (31). In short, the PDR1-NAT1 cassette was transformed into strain NRZ-2016-252, and transformed cells were plated onto YPD + 100 µg/mL nourseothricin (Jena Bioscience, Jena, Germany) and incubated for 2 days at 30°C. Transformants were verified with colony PCR using the primer pairs G1-PDR1/X2-NAT1 and G4-PDR1/X3-NAT1 to ensure correct integration of *NAT1* into the *CgPDR1* locus (Table S4). The CgERG4-ScLEU2 and CgERG3-ScHIS3 cassettes were transformed into *N. glabratus* HTL, which is auxotrophic for histidine, leucine, and tryptophan (32). Transformed cells were plated onto SDG agar containing required amino acids and incubated for up to three days at 30°C. Transformants were verified with colony PCR, and the primers used for verification are listed in Table S4. Consequently, the CgERG3-ScHIS3 cassette was transformed into HTL *erg4::ScLEU2* (*erg4*∆), the transformed cells were plated onto SDG medium with tryptophan and incubated for 3 days at 30°C. Transformants were verified with colony PCR using primers listed in Table S4.

We excised the wild-type *ERG3* allele together with the *NAT1* gene and homology regions to the *ERG3* from plasmid pSK-CgERG3^WT^-NAT1 and transformed the cassette into NRZ-2016-252, similar to the described *PDR1* deletion above. Transformants were first verified with colony PCR using the primer pairs G1-ERG3/X2-NAT1 and G4-ERG3/X3-NAT1 to ensure correct integration of *NAT1* into the *CgERG3* locus (Table S4). Additionally, we used Sanger sequencing of the G1-ERG3/G4-ERG3 PCR product to confirm the replacement of the mutated *ERG3*^Q26*^ allele with wild-type *ERG3*.

### Transcriptome analysis

Fungal cells were grown overnight in YPD at 37°C. 1 × 10^6^ cells/mL were then added to prewarmed YPD with or without 1 µg/mL AMB and grown for 1 h at 37°C. Cells were harvested by centrifugation, and total RNA was isolated as previously described (33). Library preparation and RNA-sequencing were performed by the Core Unit SysMed Würzburg. RNA quality was checked using a 2100 Bioanalyzer with the RNA 6000 Nano kit (Agilent Technologies). The RIN for all samples was >9.1. cDNA libraries were prepared from 500 ng of total RNA with TruSeq mRNA Stranded Library Prep Kit from Illumina according to the manufacturer’s instructions (1/2 volume). Libraries were quantified by Qubit Flex Fluorometer (ThermoFisher), and quality was checked using 2100 Bioanalyzer with High Sensitivity DNA and DNA 1000 Kit (Agilent). Sequencing of pooled libraries, spiked with 1% PhiX control library, was performed at ~20 million reads/sample in single-end mode with 75 nt read length on the NextSeq 500 platform (Illumina). Demultiplexed FASTQ files were generated with bcl2fastq2 v2.20.0.422 (Illumina).

RNA-seq reads were quality controlled with fastqc v0.11.9 (34) and adapter clipped with fastp v0.20.0 (35). After another quality control, reads were mapped to the reference genome ASM254v2 using the STAR v2.7.10 tool (36). Annotation count files were generated with featureCounts v2.0.6 (37). Gene-based analysis was performed with DESeq2 v1.38.3, filtering out variants with *P*_adj_ ≤0.05 and an absolute fold change ≥2 for upregulated genes and an absolute fold change ≤0.5 for downregulated genes (38). The Volcano Plot was prepared with the R package EnhancedVolcano v1.16.0.

### Gene expression analysis

An amount of 100 ng/µL of total RNA from the same conditions as used for the transcriptome analysis was the template for RT-qPCR using the Luna Universal One-Step RT-qPCR Kit with SYBR Green (New England Biolabs). Table S4 lists all used oligonucleotide primers. Gene expression was calculated with the ∆∆Ct method (39). *RDN5.8* and a control RNA (5 h YPD, 37°C) were used for normalization. Data from independent biological triplicates were compared with a two-tailed, unpaired Student’s *t*-test, and *P* values ≤ 0.05 were regarded as statistically significant.

### Growth and stress tests

For performing the growth curve experiment, the *N. glabratus* strains were grown in YPD medium overnight at 37°C. Afterward, they were diluted into fresh YPD (pre-warmed at 37°C) to an OD_600_ = 0.1. The growth curve experiments were performed for 11 h, and measurements were made after each hour. Three independent biological experiments were performed. GraphPad Prism 10.6.1 was used to visualize the growth curve and to perform Friedman testing followed by Dunn’s multiple comparison testing. Doubling times (*t*_*d*_) were calculated only for the experimental growth phase, using *t*_*d*_ = ln(2)/µ, with µ = [(ln*N*2) − ln(*N*1)]/(*t*2 − *t*1), with *N* being the measured OD_600_ value at the given timepoint (*t*).

For stress resilience testing, *N. glabratus* strains were grown in YPD medium overnight at 37°C. Out of this preculture, a suspension with 1 × 10^8^ fungal cells/mL was made with fresh YPD medium. This suspension was then used to make further dilutions in YPD medium (10^−2^, 10^−4^, and 10^−6^). Five microliters of each dilution was dropped onto YPD agar or YPD plates containing 400 µg/mL Congo Red (Sigma Aldrich, Germany), 1.5 M sodium chloride (Carl Roth, Germany), or 0.0125% SDS (Carl Roth, Germany) and incubated for 3 days at 37°C. Alternatively, YPD plates with drops of fungal cells were incubated at 42°C for 3 days.

### Assessment of *in vivo* pathogenicity in *Galleria mellonella* larvae

Prior to the inoculation with *N. glabratus*, *Galleria mellonella* larvae weighing 220–280 mg fasted for 24 h. Larvae were wiped with 70% ethanol, grouped, and placed in individual petri dishes for observation. *N. glabratus* CBS138 and NRZ-2016-252 were grown overnight at 37°C on Sabouraud dextrose agar. Fungal cells were then harvested and suspended in sterile phosphate-buffered saline (PBS). Each larva within a group was injected with 10 µL of the yeast cell suspension with 2 × 10^9^ colony-forming units/mL, resulting in the inoculation of 2 × 10^7^ yeast cells into each larva. The control group larvae were injected with sterile PBS. The infected larvae were incubated at 37°C. Larvae of the intervention groups were injected with AMB (5 mg/kg body weight) 1 h after inoculation. Larval survival, indicated by melanization and mobility, was periodically monitored during the next 7 days. Shown are the data for 20 larvae for each group, pooled from two independent biological experiments. GraphPad Prism 10.6.1 was used to visualize survival with Kaplan-Meier plots and to perform Mantel-Cox tests for curve comparison.

## Data Availability

Sequencing data are available in the National Center for Biotechnology (NCBI) Sequence Read Archive (SRA) under BioProject PRJNA1299776.

## SUPPLEMENTAL MATERIAL

Supplemental Figure Legends mbio.02731-25-s0001.docx Legends for Figures S1 and S2.

Figure S1 mbio.02731-25-s0002.tif Pdr1-mediated upregulation of efflux pumps.

Figure S2 mbio.02731-25-s0003.tif Growth of AMB resistant strains on different media and under cell wall stress conditions.

Table S1 mbio.02731-25-s0004.xlsx Sterol composition of clinical *N. glabratus* isolates.

Table S2 mbio.02731-25-s0005.xlsx Transcriptome analysis an AMB susceptible and an AMB resistant *N. glabratus* strain in response to AMB.

Tables S3-S5. mbio.02731-25-s0006.docx Strains, oligonucleotide primers, and plasmids.

ASM does not own the copyrights to Supplemental Material that may be linked to, or accessed through, an article. The authors have granted ASM a non-exclusive, world-wide license to publish the Supplemental Material files. Please contact the corresponding author directly for reuse.
