# Supplementary material for: Ergosterol-depleted clinical isolates of Nakaseomyces glabratus can develop multi-drug resistance without severe fitness defects or attenuated virulence in an invertebrate infection model
Source: mBio. 2026 May 20;17(6):e02731-25. doi: 10.1128/mbio.02731-25 (PMC13251370; doi:10.1128/mbio.02731-25)
Supplement: Supplemental Figure Legends — Legends for Figures S1 and S2. [file mbio.02731-25-s0001.docx]

**Figure S1. Deletion of *PDR1* led to downregulation of efflux pump genes in NRZ-2016-252 and increased susceptibility to FLU.**

(A) Expression of efflux pump genes in NRZ-2016-252 and NRZ-2016-252 *pdr1*∆ after 1 h growth in YPD medium at 37°C. Four biological independent samples from each strain were used for isolation of total RNA and RT-qPCR. Gene expression was normalized against CBS138 (1h YPD, 37°C) and the *RDN5.8* gene. Asterisks indicate significant changes in gene expression between the two *N. glabratus* strains (two-tailed, unpaired student’s *t*-test, p ≤ 0.05). (B) The indicated strains were plated onto RPMI1640 medium, and E-test stripes for AMB, ANF and FLU were applied. Pictures were taken after an incubation for 48h at 37°C.

**Figure S2. Growth of AMB^R^ isolates under different growth conditions including high temperature and cell wall stress.**

The indicated *N. glabratus* strains were grown overnight in YPD at 37°C. 5µl of 1x10^8^, 1x10^6^, 1x10^4^ and 1x10^2^ cells /ml of these overnight cultures were spotted onto the indicated plates (from top to bottom). (A) Strains were grown for 3 days at either 37°C or 42°C on YPD, SDG or RPMI 1640 medium prior to microscopy. (B) Strains were grown at 37°C on either YPD or SDG containing 25 µg/ ml Calcofluor White or 200 µg/ ml Congo Red. Pictures were taken after a 3-day incubation period. Plates shown in (A) and (B) are representatives of three biological independent experiments.
