# Supplementary figures and images for: Ergosterol-depleted clinical isolates of Nakaseomyces glabratus can develop multi-drug resistance without severe fitness defects or attenuated virulence in an invertebrate infection model
Source: mBio. 2026 May 20;17(6):e02731-25. doi: 10.1128/mbio.02731-25 (PMC13251370; doi:10.1128/mbio.02731-25)

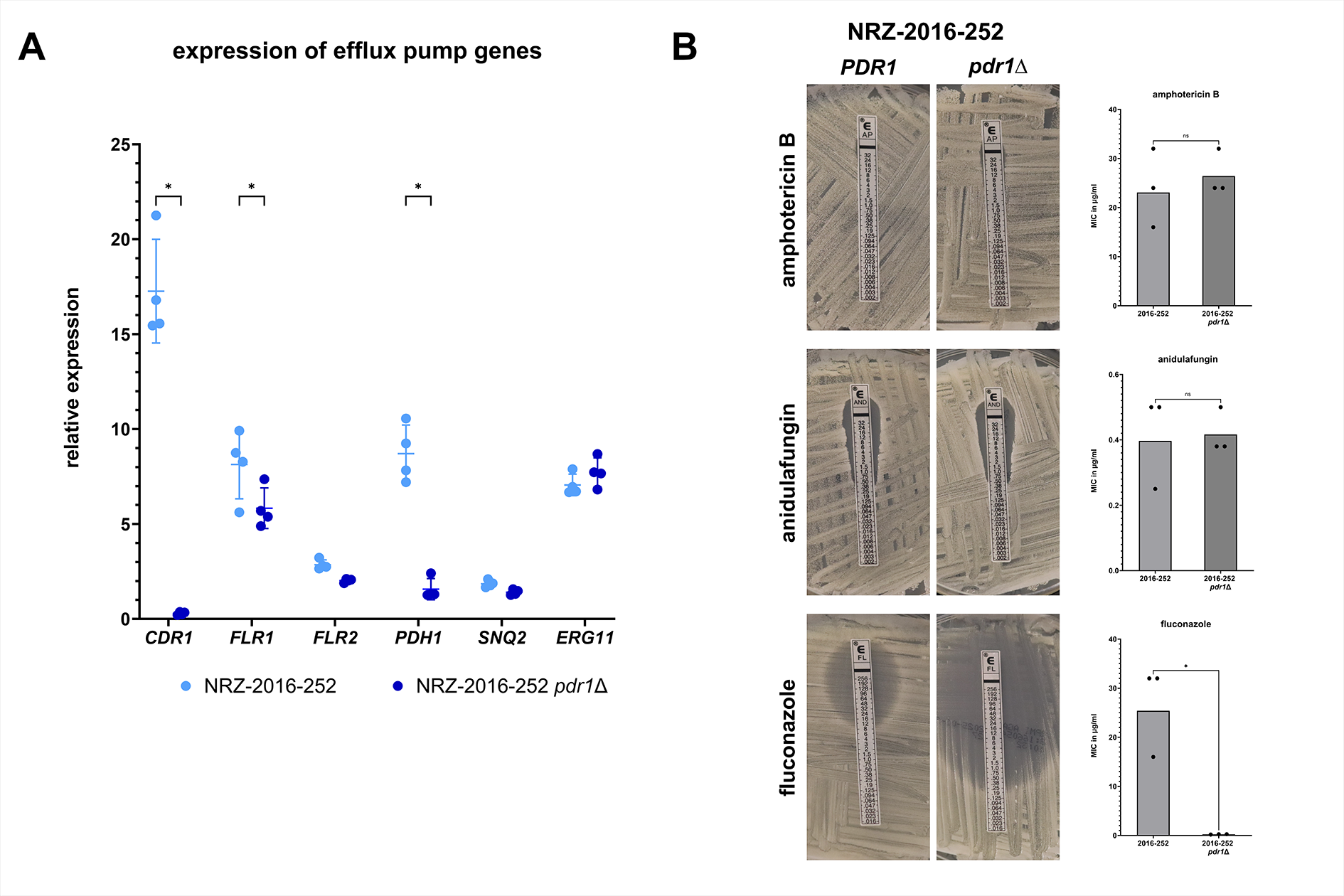

Supplement: Figure S1 — Pdr1-mediated upregulation of efflux pumps. [file mbio.02731-25-s0002.tif]

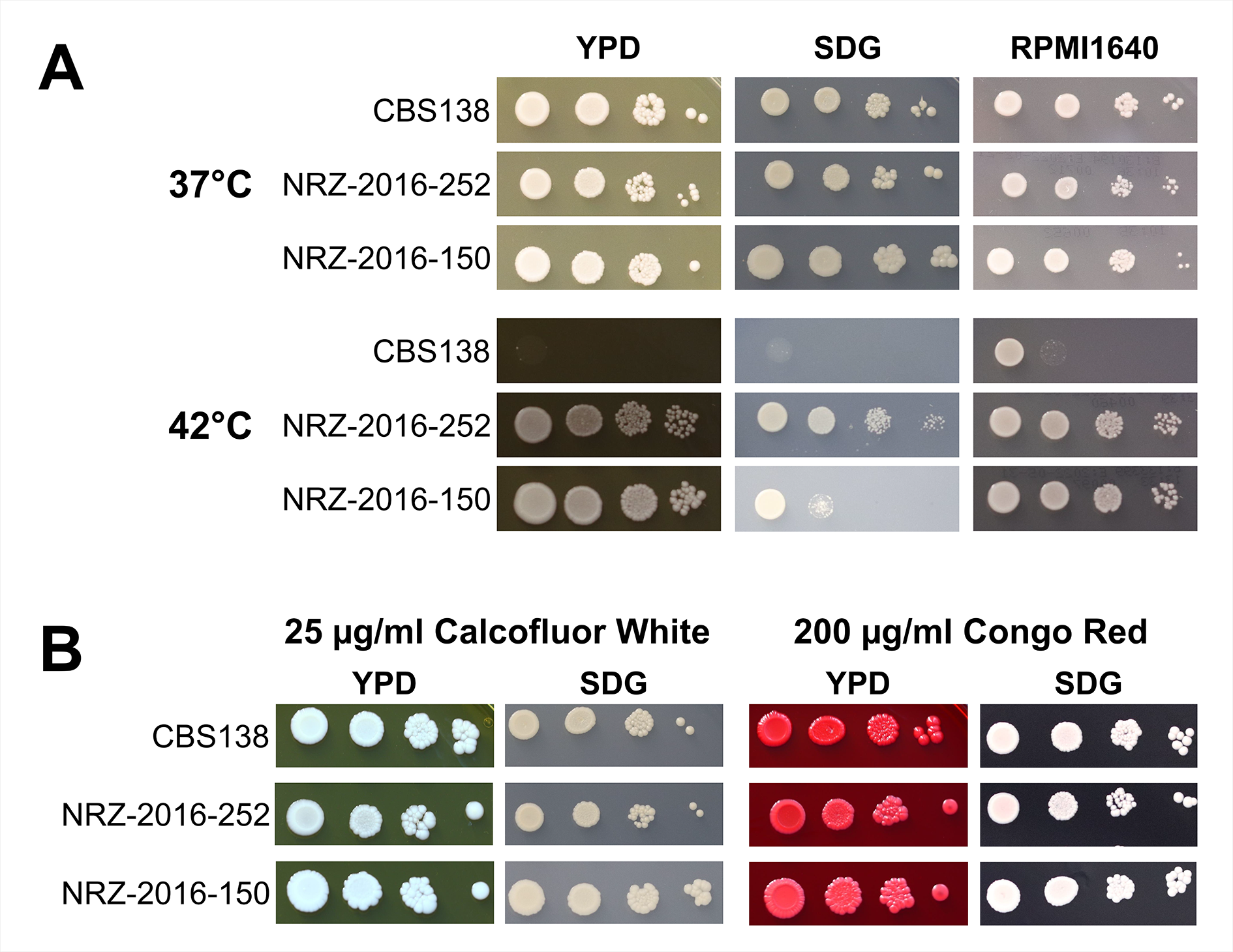

Supplement: Figure S2 — Growth of AMB resistant strains on different media and under cell wall stress conditions. [file mbio.02731-25-s0003.tif]
